# Characterization of *O*-Glycosylation and *N*-Glycosylation in Bispecific Antibodies and Its Importance in Therapeutic Antibody Development

**DOI:** 10.3390/ph18101538

**Published:** 2025-10-13

**Authors:** Maoqin Duan, Luyun Guo, Zhen Long, Yongbo Ni, Yalan Yang, Jialiang Du, Meng Li, Jialing Zhang, Tao Tang, Chuanfei Yu, Lan Wang

**Affiliations:** 1 Key Laboratory of the Ministry of Health for Research on Quality and Standardization of Biotech, Division of Monoclonal Antibody Products, National Institutes for Food and Drug Control, Beijing 102629, China; duanmaoqin@nifdc.org.cn (M.D.); guoluyun@nifdc.org.cn (L.G.); tangtao_scu@163.com (T.T.); 2 ThermoFisher Scientific Corporation, Beijing 100080, China; longzhen8866@126.com

**Keywords:** BsAbs, *O*-glycosylation, *N*-glycosylation, MS, HILIC-HPLC, HPAEC-PAD, ELISA, mannose receptor

## Abstract

**Background/Objectives**: This study comprehensively characterized the *O*- and *N*-glycosylation profiles of bispecific antibodies (BsAbs) via advanced analytical techniques to evaluate their structural and functional implications. **Methods**: High-resolution MS revealed *O*-xylosylation at Ser468 within the (G4S)4 linker peptide, which was identified as xylose with a molecular weight of 132.042 Da. HILIC-HPLC analysis of *N*-glycosylation revealed glycan species engineered to eliminate Fc effector functions. *O*-glycosylation analysis via β-elimination followed by high-performance anion-exchange chromatography with pulsed amperometric detection (HPAEC-PAD) identified xylose as the predominant glycan. **Results**: *O*-xylosylation does not affect the binding of BsAbs to either antigen Programmed Death-1 (PD-1) or Vascular Endothelial Growth Factor (VEGF). Notably, *O*-xylosylation interactions with mannose receptor represent the first discovery highlighting potential immunomodulatory roles. **Conclusions**: This study highlights the critical importance of monitoring comprehensive glycosylation characterization during the development of BsAb with (G4S)n linkers to ensure optimal therapeutic efficacy, safety, and reduced immunogenic potential.

## 1. Introduction

The use of protein biotherapeutic drugs has gained widespread interest in the treatment of various diseases. Antibodies are a major class of beneficial and effective drugs that have been developed as treatments for a variety of therapeutic areas. The substantial number of antibodies entering clinical studies and approval has been well-reported. Monoclonal antibodies (mAbs) commonly include the immunoglobulin G (IgG) 1, IgG2 and IgG4 subclasses [1,2,3]. With the advancement of technology, bispecific antibodies (BsAbs) have emerged as a significant innovation in biopharmaceuticals that may have different heavy chains and light chains, and other IgG-based molecules in alternative formats, such as those with the addition and removal of the antigen-binding fragment (Fab) arm, have also been created to achieve advantageous properties, such as the ability to interact with multiple therapeutic targets. In cancer therapy, BsAbs can achieve high target specificity by simultaneously binding to one tumor-associated antigen and recruiting tumor-killing agents (e.g., T cells) via a second binding site [4,5,6], thereby enhancing specificity, minimizing off-target toxicities, and synergistically modulating anti-tumor immunity and the tumor microenvironment. Compared with the combination of two monoclonal antibodies, BsAbs represent the physical integration of dual specificities, demonstrating superior binding efficacy, reducing the risk of drug resistance, and enabling unique biological functions such as bridging tumor cells and T cells to achieve precise cytotoxicity. Full-size antibodies consisting of several polypeptide chains, are typically synthesized in eukaryotic, mainly mammalian, cell lines (Chinese hamster ovary and human embryonic kidney cells 293). Such systems can provide correct folding, post-translational modifications, and glycosylation according similar to those in human cells.

BsAbs are being developed to form various formats (e.g., bispecific T-cell engagers, dual-affinity retargeting proteins, Knobs-and-holes, and double-variable domains) [7]. The most frequently used linkers consist of Gly-Gly-Gly-Gly-Ser (G4S) amino acid repeats, as they are structurally flexible and protease resistant [8]. For example, otlertuzumab, which targets CD37, is composed of single-chain variable fragments (scFvs) attached to the N-terminal residues of the Fc domain [9]. The VH and VKAPPA moieties of the scFv are conjugated via a (G4S)5 linker peptide. The fusion protein cergutuzumab amunaleukin includes a (G4S)3 linker peptide [10]. Glycosylation analysis of the scFv protein, which was linked to the C-terminal residues of the heavy chains (H-chains) of the monoclonal antibody (mAb) via a G4S linker peptide, revealed three key findings: (1) *O*-xylosylation is a common posttranslational modification (PTM) for (G4S)n (n > 2) linkers, (2) *O*-xylosylation may be related to the GSG motif, and (3) The total xylosylation per linker increases with the number of GSG motifs [8,11]. Notably, unanticipated *O*-glycosylations have been reported in Fc-fusion proteins and BsAbs produced in mammalian cell expression systems.

The majority of protein biotherapeutic drugs display more posttranslational modifications (PTMs). One of the notable and best-known PTMs significantly affecting protein conformation and cellular functions is glycosylation. Glycosylation is not only essential for biological functions (or effector functions) but also influences the physicochemical properties of proteins, such as conformation, stability, solubility, safety and efficacy [12,13,14,15]. *N*-glycosylation is the major type of posttranslational modification, and the glycan is attached to the side chain of an Asn residue located in the consensus sequence of Asn-X-Ser/Thr (note that position 2 [X] can be any amino acid except Pro; occasionally, position 3 can be a Cys residue) [16,17,18]. The function of *N*-glycans on mAbs has been widely researched. The modification of *N*-glycans can affect the interaction of the Fc segment with Fc receptors (FcγRs), which are responsible for antibody-dependent cell-mediated cytotoxicity (ADCC). Reducing the level of fucosylation on the *N*-glycan of an antibody can greatly increase ADCC activity [19,20,21,22]. Modern analytical techniques for *N*-glycosylation are well established, enabling rapid characterization of *N*-glycosylation, thereby offering critical insights and traceability to support regulatory compliance [23,24], including chromatography methods and electrophoresis techniques. Reverse-phase (RP) liquid chromatography and hydrophilic interaction liquid chromatography (HILIC) are applied to achieve proper *N*-glycosylation separation [25]. Label-free native *N*-glycans can be analyzed via HPAEC-PAD, which has good selectivities for isomeric structures and improved retention of charged glycans [26]. Mass spectrometry (MS) systems with matrix-assisted laser desorption ionization (MALDI) and electrospray ionization (ESI) are typically used for glycan profiling [27]. Surely, more reliable and sensitive methods are needed.

Glycosylation can be comprehensively researched in a wide range of biopharmaceuticals. The other major type of posttranslational modification involved in glycosylation is *O*-glycosylation. The glycan is attached to the side chain of a Ser or Thr residue. *O*-glycosylation encompasses a variety of modifications determined by the innermost (termed the reducing end) monosaccharide and leads to a more heterogeneous pool of oligosaccharides with different initiation sugars and linkages. *O*-glycosylation was found to impose critical impacts on drug immunogenicity, protein secretion and protective immunity against cancer, and maintenance of normal development and physiology—as well as other aspects that remain incompletely understood [28]. Additionally, *O*-glycosylation of biopharmaceuticals in analytical strategies faces limitations, posing daunting challenges to development and regulatory compliance. A universal *O*-glycanase that is capable of liberating *O*-glycans with more sophisticated branching and elongation is still unclear. β-Elimination, as a chemical release of *O*-glycans, is a common approach [29,30,31]. This reductive elimination method has been shown to be the most reliable method for preserving *O*-glycan structures. However, a severe disadvantage is that the reduced sugar prevents further glycan labeling through the reducing end hydroxyl group and eliminates the possibility of utilizing this end for fluorescence-based detection and quantitation. Therefore, the label-free detection method of HPAEC-PAD uses a commonly sensitive quantitation without the need for a fluorophore [32]. Hydrazinolysis is another nonreductive method for *O*-glycan release without excessive peeling. However, the use of highly harmful hydrazine still represents the worst flaw of this method. All chemical release methods lack the desirable specificity for the release of *O*-glycosylation compare to *N*-glycosylation [33]. The released *O*-glycosylation can be analyzed via HILIC, and porous graphitic carbon (PGC) can be used for glycan separation [34]. MALDI-MS and ESI-MS are the two most prevalent MS techniques for *O*-glycosylation analysis [35,36].

**Figure 1:**
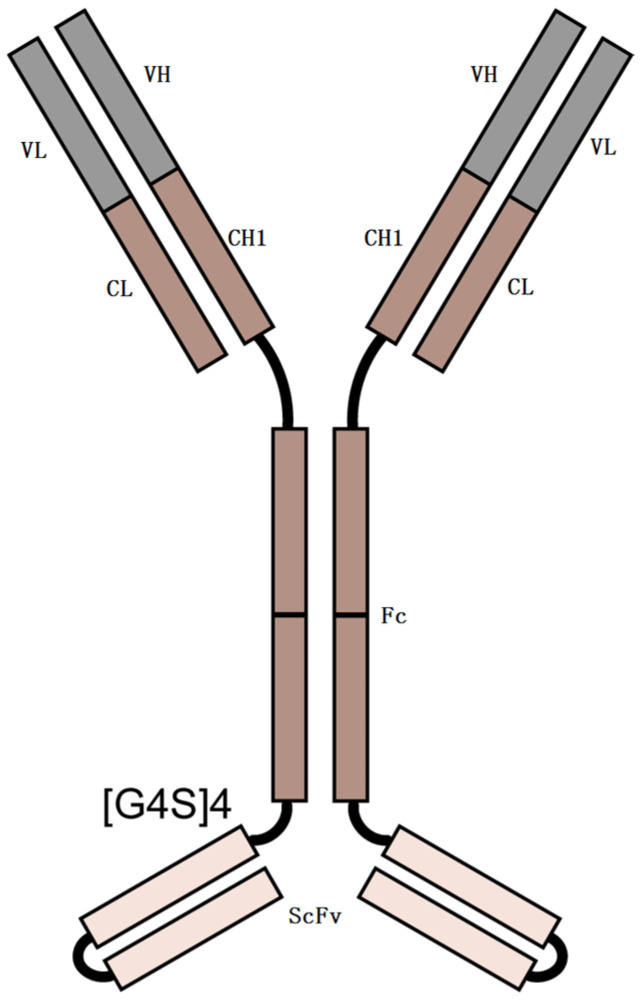
Schematic representation of BsAb. Linker = (G4S)4.

The majority of biopharmaceutical drugs are monoclonal antibodies of the IgG isotype. These IgG molecules are not *O*-glycosylated, with only one *N*-glycosylation located in each Fc domain. However, *O*-glycosylation is prevalent in many other therapeutic protein classes, including bispecific antibodies (BsAbs) [37]. For instance, the BsAb we studied, shown in Figure 1, has *O*-glycosylation. BsAb is an engineered antibody that can neutralize two different human antigen proteins (PD-1 and VEGF). BsAb is composed of a human IgG (mAb1) and the variable domains of the second human IgG (mAb2). The C-terminus of the heavy chain of mAb1 is linked to the heavy-chain variable domains (VH) of mAb2 by a (GlyGlyGlyGlySer)4 [(G4S)4] linker. The peptide linker (G4S)4 is located at positions 454–473.

This study demonstrates *O*-glycosylation occurring on BsAbs containing linkers composed of G4S amino acid repeats. An in-depth understanding of the *O*-glycosylation status of BsAbs will shed light on the structure-function relationship of *O*-glycosylation. Preliminary investigations were conducted to assess the structural and functional implications of *O*-glycosylation for both target binding activity and Fc effector functions, suggesting the criticality of *O*-glycosylation translational modifications. Risk-based regulatory approval approaches still require the elucidation of *O*-glycosylation patterns routinely and quantitatively, particularly critical for maintaining a consistent product quality profile of both primary drugs and biosimilars to minimize potential drifts in efficacy and safety profiles.

## 2. Results

### 2.1. Characterization of N- and O-Glycosylations

#### 2.1.1. Intact Protein and HC-LC Analysis

**Figure 2:**
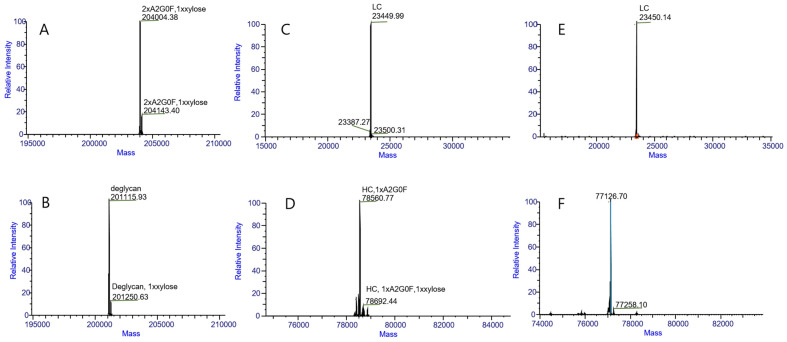
LC/MS intact mass measurement results for intact proteins (**A**,**B**), light chains (**C**,**E**) and heavy chains (**D**,**F**). (**A**) was obtained with a *N*-glycosylation protein of about 204,004.38 Da, to further localize the 139 Da modification within the protein about 204,143.40 Da. (**B**) was obtained with *N*-deglycosylation protein of about 201,115.93 Da, to further localize the 135 Da modification within the protein about 201,250.63 Da. (**C**) was obtained with a *N*-glycosylation protein with LCs and no modification within the LC. (**D**) was obtained with a *N*-glycosylation protein with HCs of about 78,560.77 Da, to further localize the 132 Da modification within the protein at about 78,692.44 Da. (**E**) was obtained with a *N*-deglycosylation protein with LCs and no modification within the LC. (**F**) was obtained with *N*-deglycosylation protein with HCs of about 77,126.70 Da, to further localize the 132 Da modification within the protein of about 77,258.10 Da. These data demonstrate that the protein contains a modification of approximately 132 Da, which persists after *N*-deglycosylation treatment.

Intact protein and HC-LC analyses were performed on two types of samples: *N*-deglycosylation and *N*-glycosylation proteins. Both *N*-glycosylation (Figure 2A) and *N*-deglycosylation (Figure 2B) protein samples detected a component that exhibited a molecular weight difference of approximately 132 Da compared with the main component. This observation indicates the presence of a modification with a molecular mass of approximately 132 Da in the protein and critically demonstrates that this modification is resistant to *N*-deglycosylation treatment. The results in Figure 2A also demonstrate that the molecular weight of the protein aligns with the theoretical expectations (201,120.52 Da). A comparison of the data in Figure 2A,B suggests that the predominant *N*-glycoform of this protein is likely A2G0F. To further localize the 132 Da modification within the protein, we conducted HC-LC analysis. As shown in Figure 2C–F, the modification of light chains (LC) was undetectable but was consistently observed on heavy chains (HC) in both the *N*-deglycosylation and *N*-glycosylation samples, demonstrating its specific localization to HC. Comparative analysis of the data in Figure 2D,F reaffirmed that this modification is resistant to *N*-deglycosylation.

#### 2.1.2. Peptide Mapping Analysis

**Figure 3:**
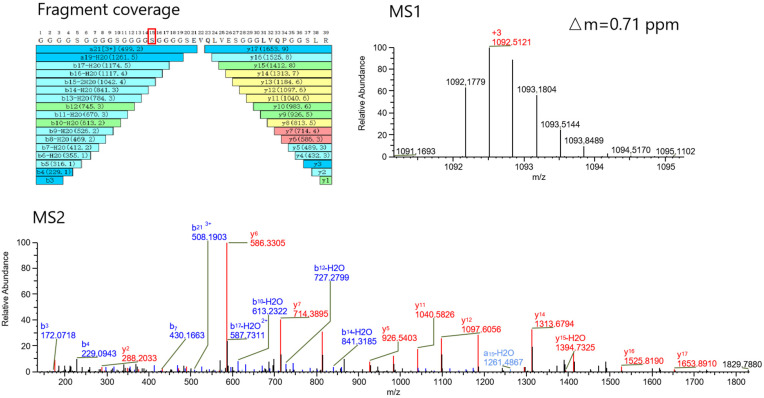
MS1 and MS2 spectra of xylose-modified peptides. High sequence coverage of the resulting peptides and identified Ser 468 in red box as the modification site bearing the 132.042 Da group was achieved. This modification is tentatively assigned as xylose moiety (C_5_H_10_O_5_, theoretical mass: 132.042 Da).

In the modification profiling, we identified Ser 468 as the modification site bearing the 132 Da group, with an exact mass of 132.042 Da (Figure 3). This modification is tentatively assigned as xylose conjugation on the basis of its mass correspondence to the xylose moiety (C_5_H_10_O_5_, theoretical mass: 132.042 Da). From peptide mapping, we also determined that the modification ratio of xylose was 5.98%. This ratio agrees well with the results detected at the intact level (9.71%) and the HC-LC level (6.16%).

#### 2.1.3. HPAEC-PAD Analysis of *O*-Glycosylation

**Figure 4:**
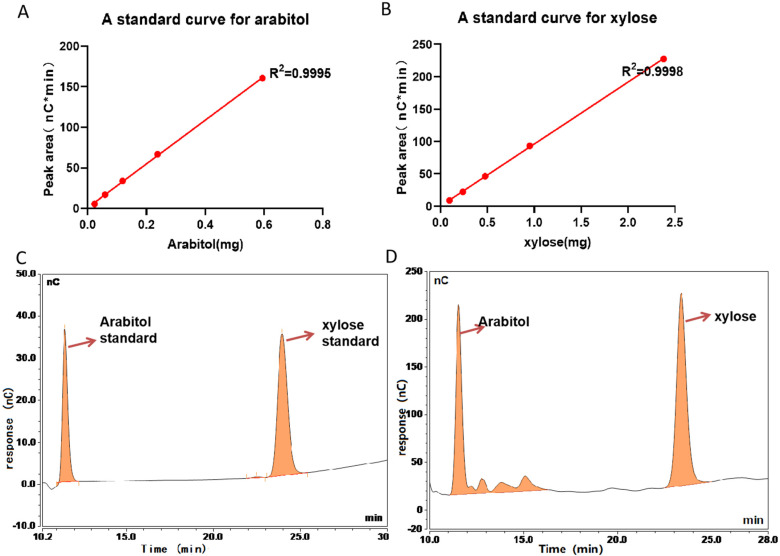
HPAEC/PAD chromatogram of the β-elimination extract of BsAbs. (**A**) Calibration curve for standard Arabitol, Y = 122.5*X − 0.5715 (R^2^ = 0.9994). (**B**) Calibration curve for standard xylose, Y = 194.9*X + 0.1169 (R^2^ = 0.9998). (**C**) The Standards of 0.119 μg of Arabitol standard and xylose standard. (**D**) HPLC analysis identified xylose as a major glycan constituent in BsAbs. The chromatograms three separate experiments were superimposable at this resolution.

If the modification of 132.042 Da is xylose, it can be released by β-elimination and detected via the HPAEC-PAD method. Arabitol was added to the test samples as an internal spiking standard. The contents of xylose were quantitatively estimated via this method. A calibration curve was obtained for xylitol and xylose at 5 different concentrations (0.0238, 0.0595, 0.119, 0.238, and 0.595 μg), and the absorbance of each sample was measured via pulsed amperometric detection to obtain the following linear equations: Y = 122.5 × X − 0.5715 (R^2^ = 0.9994) and Y = 194.9 × X + 0.1169 (R^2^ = 0.9998), respectively (Figure 4A,B). xylose was identified as a major constituent of the extracts from the chromatographic analysis of the BsAbs according to the described procedure. The Standards of 0.119 μg of sugars was presented in Figure 4C. Except for those visible in the chromatograms (Figure 4D), significant signals of *O*-glycosylation compounds were not observed.

#### 2.1.4. *N*-Glycosylation

**Figure 5:**
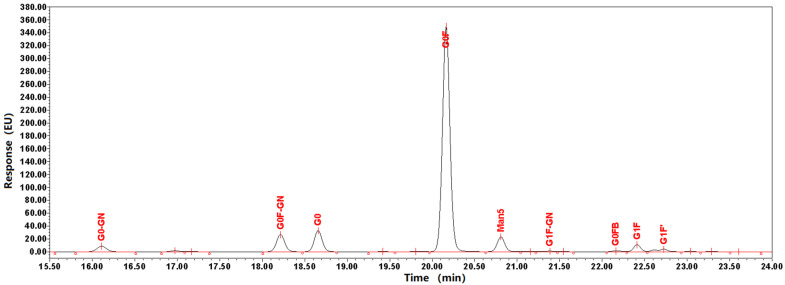
Glycan profiles of BsAbs. Glycans were cleaved with PNGaseF and isolated prior to labeling with 2-aminobenzamide. The profiles were normalized relative to the height of the G0F peak. The profiles of three separate experiments were superimposable at this resolution. The same sample repeated the same experiment 3 times.

*N*-glycosylation is also important modification of antibodies. After the analysis of molecular weight, light chain, heavy chain, peptide sequence coverage and *O*-glycosylation, *N*-glycosylation were detected by HILIC-HPLC. As shown in Figure 5, galactose-zero (G0) with core fucosylation (F) (G0F) was the most abundant *N*-glycan (76%). In addition to the glycoforms G0F, G0-GlcNAc, G0F-GlcNAc, G0, G1F, and Man5 also presented a low abundance (<10%), exhibiting negligible effects on Fc region function.

### 2.2. ELISA-Based Analysis of the Effects of O-Glycosylation on BsAbs

**Figure 6:**
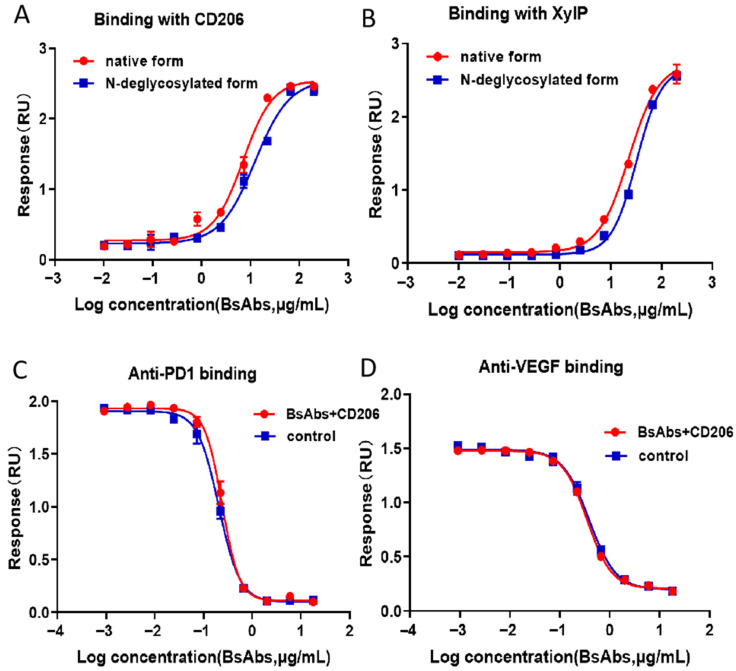
Results of the ELISA-based assay profiles. (**A**) Native BsAbs and enzymatically *N*-deglycosylated BsAbs can bind to MR, and the EC50 values were approximately 5 μg/mL and 9 μg/mL, respectively. (**B**) Native BsAbs and an enzymatically *N*-deglycosylated BsAb can bind XylP, and the EC50 values were approximately 23 μg/mL and 32 μg/mL, respectively. (**C**) Under identical experimental conditions, BsAbs binding both MR-binding and non-MR-binding BsAbs targeting PD-1 showed no significant differences in binding affinity, demonstrating comparable four-parameter logistic curves. (**D**) Under identical experimental conditions, BsAbs binding both MR-binding and non-MR-binding BsAbs targeting VEGF showed no significant differences in binding affinity, demonstrating comparable four-parameter logistic curves. The error bars represent the means ± SDs from three experiments.

ELISA-based binding studies were performed to evaluate the interaction between the MR and two BsAb variants: the native form and an enzymatically *N*-deglycosylated form. Positive binding signals were observed, and the absence of *N*-glycosylation did not affect binding activity with the MR. Due to the presence of mannose molecules in *N*-glycosylation, both *N*- and *O*-glycosylation in native form of antibodies bind to mannose receptors (Figure 6A). Quantitative analysis revealed a half-maximal effective concentration (EC50) for the binding of BsAbs to MR, thereby justifying further mechanistic investigations. XylP can bind the native form and an enzymatically *N*-deglycosylated form of BsAbs (Figure 6B), with a dose–response curve at different concentrations of BsAbs. The intact bispecific antibody (36 μg/mL starting concentration) and antigen (720 ng/mL) were mixed at a 1:1 volume ratio for coincubation. In parallel control experiments, antibodies at identical concentrations were incubated with dilution buffer instead of antigen. Binding assays were independently performed for both antigen targets PD1 and VEGF (Figure 6C,D). Compared with the parallel control, the bioassay targeting PD-1 of the BsAb binding to the MR retained approximately 96% of its binding activity without affecting its binding to the target PD-1. Compared with the parallel control, the bioassay in which the BsAb binds to the MR retained approximately 94% of its binding activity without affecting its binding to the target VEGF.

## 3. Discussion

Prior to exploring the effects of *N-* and *O*-glycosylation on bioactivity, a comprehensive characterization of BsAb, including sequence coverage, molecular weight, and posttranslational modifications (PTMs), such as *N*- and *O*-glycosylation, are key. In this study, the protein was characterized via LC-MS at three levels: intact protein, Heavy Chain-Light Chain (HC-LC), and peptide. After intact analysis confirmed the existence of a 132 Da modification, peptide mapping was used to detect the accurate mass and location of this modification. Since both *N*-glycosylation and the 132 Da modification reside on the heavy chain (HC), colocalization of these modifications on the same peptide segment would complicate site-specific assignment of the 132 Da modification. To eliminate interference from *N*-glycosylation, *N*-glycosylation removal was performed prior to proteolytic digestion. The achieved high sequence coverage of the resulting peptides, combined with intact mass analysis results, confirmed both the sequence accuracy and structural integrity of the protein. For *O*-glycosylation profiling, β-elimination followed by HPAEC-PAD analysis was additionally employed, further confirming the presence of *O*-glycosylation. For *N*-glycosylation profiling, with PNGaseF and isolated prior to labeling with 2-aminobenzamide by HILIC-HPLC analysis was additionally employed G0F that without CDC and ADCC activities.

Various studies have shown that animal lectins have diverse functions, ranging from cell–cell interactions to immune responses [38]. The diverse functions of lectins are due to their ability to recognize and differentiate among various glycan molecules. The primary function of lectins is to bind with carbohydrates and glycoconjugates to decode their information and mediate various functions, including intercellular interactions, cell signaling, and immune responses [39]. Different types of C-type lectins, including Dectin-1, Dectin-2, and Mannose receptors (MR), have been identified [40]. CD206 is the prototype member of the MR family of proteins [41,42] and contains a functional carbohydrate-binding receptor (CR) domain [43]. In MR, the CR domain binds sulfated carbohydrates, particularly galactose or GalNAc, which are sulfated at position 3 or 4 [43,44]. An in-depth understanding of the *O*-glycosylation status of these drug substances will shed light on the structure–function relationship of *O*-linked sugars, which may lead to the identification of functionally favorable *O*-glycosylation structures to improve drug efficacy and safety profiles. In particular, *O*-xylose can bind to the mannose receptors of C-type lectins. The MR is closely associated with immunity, infection, and closely related processes. For a detailed understanding of *O*-glycosylation function, the risk-based approach for regulatory approval still warrants the elucidation of the *O*-glycosylation pattern routinely and quantitatively. Unintentional glycosylation may affect the efficacy and safety of BsAbs by altering their biological activities and immunogenicity. For therapeutic antibodies with CDC and ADCC activities, close monitoring of glycoform profiles and alterations is essential.

Given the above analysis, we will implement ongoing surveillance for potential variations arising from modifications to the manufacturing process or production site of this BsAbs and antibodies with homologous structural frameworks. Nevertheless, as BsAbs lacks ADCC and CDC functions, it is still critical to closely monitor and analyze glycoform profiles. This is highly beneficial for evaluating process stability and product consistency. While BsAbs with novel structures must require exhaustive *O*-glycosylation analysis, those with homologous frameworks possessing CDC and ADCC functions necessitate comprehensive characterization to ensure their quality, efficacy, and safety.

## 4. Materials and Methods

### 4.1. Chemicals and Materials

BsAbs (10 mg·mL^−1^) were collected by the National Institutes for Food and Drug Control (Beijing, China) and stored at 4 °C before use. Trypsin and PNGaseF were obtained from Promega (Madison, WI, USA). Ammonium bicarbonate (NH_4_HCO_3_), dithiothreitol (DTT), 2-aminobenzamide, sodium cyanoborohydride, dimethyl sulfoxide (DMSO), acetic acid, acetonitrile, ammonium formate, formic acid, anhydrous ethanol, sodium acetate, NaOH, sodium borohydride, iodoacetamide (IAM), albumin bovine serum (BSA) and xylose were purchased from Sigma-Aldrich (St. Louis, MO, USA). Formic acid (FA) was purchased from Thermo Fisher (Sunnyvale, CA, USA). Acetonitrile 10 kDa and 30 kDa centrifugables were obtained from Merck (Billerica, MA, USA). High-purity water was prepared with a Milli-Q system (Bedford, MA, USA). Hydrochloric acid, ammonium bicarbonate, sulfuric acid and glacial acetic acid were purchased from Sinopharm Chemical Reagent Co., Ltd. (Shanghai, China). Arabitol was obtained from Acros Organics. (Geel, Belgium). AG50W-X8 resin was obtained from Bio-Rad. (Hercules, CA, USA). Antigen-Programmed Cell Death Protein 1-human Fc fusion protein (PD-1-hFc), ligand protein Programmed Death-Ligand 1-murine Fc fusion protein (PD-L1-mFc), Vascular Endothelial Growth Factor with Histidine tag (VEGF-His) and Vascular Endothelial Growth Factor Receptor 2 Extracellular Domain-murine Fc-Biotinylated (VEGFR^2^-ECD-mFc-bio) were collected by the National Institutes for Food and Drug Control (Beijing, China) and stored at the optimal temperature before use. Horseradish peroxidase-conjugated AffiniPure goat anti-human IgG and Fcγ fragments were purchased from Jackson ImmunoResearch (Lancaster, PA, USA). Streptavidin-horseradish peroxidase was purchased from Kirkegaard & Perry Laboratories (Gaithersburg, MD, USA). Recombinant human CD206 protein (His tag) was purchased from SinoBiologica (Beijing, China). Phosphate-buffered saline (PBS-20X) and phosphate-buffered saline with Tween 20 (PBST-20X) were purchased from Cell Signaling Technology (Danvers, MA, USA). Tetramethylbenzidine (TMB) substrate solution was purchased from Surmodics IVD, Inc. (Eden Prairie, MN, USA).

### 4.2. Sample Preparation

#### 4.2.1. Pretreatment for Intact Protein, HC-LC Chain, *N*-Glycan and Peptide Mapping Analysis

A 50 mmol/L ammonium bicarbonate solution and a 50 mmol/L ammonium formate solution were prepared. A mixture of dimethyl sulfoxide (DMSO) and acetic acid (7:3, *v*/*v*) was prepared by vortex mixing DMSO and acetic acid to obtain a homogeneous DMSO-acetic acid mixture. An appropriate amount of 2-aminobenzamide (2-AB) was dissolved in a DMSO-acetic acid solution at a mass ratio of 1:20 (mg/μL) to yield a 2-AB solution. Subsequently, sodium cyanoborohydride was added to the 2-AB solution at a mass ratio of 1:16.7 (mg/μL) and thoroughly mixed to obtain the final labeling reagent. The reagents must be freshly prepared before use. The sample was diluted with 50 mmol/L ammonium bicarbonate solution to a concentration of 2 mg/mL. Then, 100 μL of the diluted 2 mg/mL solution was mixed with 4 μL of PNGase F and incubated in a water bath at 37 °C for 20 h to complete the enzymatic digestion. The *N*-deglycosylated protein sample was subjected to ultrafiltration via an ultrafiltration membrane at 21,000× *g* for 10 min. The filtrate was collected for derivatization to facilitate *N*-glycan analysis. The proteins retained on the membrane were reconstituted with water and split into three aliquots. One aliquot was used for *N*-deglycosylation molecular weight determination, another aliquot was reduced with DTT and subjected to HC-LC analysis after deglycosylation, and the third aliquot was digested with trypsin and subsequently analyzed by peptide mapping.

The filtrate was dried via a vacuum centrifugal concentrator (temperature maintained below 28 °C to prevent sialylation). The dried sample was reconstituted with 100 μL of 2-AB fluorescent labeling reagent, thoroughly mixed, and incubated at 65 °C for 3 h. The resulting mixture was subsequently used for *N*-glycan analysis.

#### 4.2.2. Pretreatment for HPAEC-PAD Analysis

Arabitol stock solutions of 10 mg/mL and Xylose stock solutions of 10 mg/mL and diluted them to obtain 0.25 mg/mL were prepared. We mixed 250 µL of Arabitol (0.25 mg/mL) and 250 µL of xylose (0.25 mg/mL) with 4750 µL of Milli-Q water to obtain working Arabitol and Xylosel Calibration Standard Mix (0.0119 mg/mL). In the next step, A calibration curve standard mixture with dilution factors of 25, 10, 5, and 2.5 and undiluted were prepared. Moreover, 50 µL of Arabitol (0.25 mg/mL) with 950 µL of Milli-Q water to acquire the working Arabitol internal spiking standard were mixed (0.0125 mg/mL). The proper amount of test sample, approximately 5 mg, was adjusted to 800 µL after the sample buffer was exchanged with water. The volume of each 500 µg sample was 100 µL to spike 50 µL of 0.0125 mg/mL internal spiking standard, which added 250 µL of 37.8 mg/mL NaBH4 in 0.1 N NaOH into the sample and the blank. All the tight tubes were placed in a metal bath at 45 °C for 16–20 h in a gray environment. When the samples were chilled in the chemical hood, 50 µL of 4 N cold acetic acid was slowly added to neutralize the samples, which inevitably minimizes foaming. Freshly prepared AG50W-X8 resin to desalt the glycan and clean it repeatedly were used. All the tubes were supplemented with 750 µL of methanol to dry in a speedvac with the temperature set to 60 °C at least 3 times, or no white residue remained. All *O*-glycan samples were transferred to HPLC vials.

### 4.3. LC Conditions

#### 4.3.1. LC Conditions for Intact Protein, HC LC Chain and Peptide Mapping Analysis

In addition to *N*-glycan analysis, all other analyses were performed on an LC-MS system configured with a Thermo Fisher Scientific™ Vanquish™ Flex UHPLC (Waltham, MA, USA) and a Q Exactive™ Plus mass spectrometer (Waltham, MA, USA) equipped with a heated electrospray ionization (HESI) interface. For intact and HC-LC chain analysis, a Mabpac RP (2.1 × 50 mm, 4 µm) column was used. For peptide mapping, an ACQUITY UPLC BEH peptide C18 (2.1 mm × 150 mm, 1.7 μm) from Waters (Milford, MA, USA) was used. Except for the columns used for peptide mapping, all the instruments and columns used were from Thermo Fisher (Sunnyvale, CA, USA). The LC conditions for intact protein, HC-LC chain, and peptide mapping analysis are shown in Table 1.

#### 4.3.2. LC Conditions for *N*-Glycosylation

*N*-glycopeptide analysis with an HPLC system with HILIC using an ACQUITY UPLC BEH glycan column (2.1 mm × 150 mm, 1.7 μm) from Waters (Milford, MA, USA) were conducted. The LC conditions for *N*-glycan analysis are shown in Table 2.

#### 4.3.3. HPAEC-PAD Analysis

*O*-glycopeptide analysis with the HPAEC System with PAD, using an analytical Dionex CarboPac MA1 column (4 mm × 250 mm, 7.5 µm), a guard Dionex Amino Trap column (3 × 30 mm, 6.5 µm) and a trap Dionex Borate Trap column (4 × 250 mm, 20 µm) from Thermo Fisher (Sunnyvale, CA, USA) were conducted.

The HPLC-PAD conditions for *O*-glycan analysis are shown in Table 3.

### 4.4. MS Parameters

The MS parameters are shown in Table 4.

### 4.5. Enzyme-Linked Immunosorbent Assay (ELISA)

The recombinant human mannose receptor C-type 1 (MRC1/CD206) protein (His tag) and recombinant human xylose transporter protein (XylP, His tag) to 100 ng/mL were diluted, added 100 μL per well to a 96-well high-binding plate, sealed the plate with sealing film, incubated it at 4 °C for 24 h, washed the 96-well plate three times with PBST and blocked it with 1% BSA in PBS for 1 h. After washing three times with PBST, the BsAbs (starting at a concentration of 100 μg/mL at a 1:3 dilution ratio, 100 μL/well) were cocultured in a 96-well plate for 60 min. After washing three times with PBST, 100 μL of horseradish peroxidase-conjugated AffiniPure goat anti-human IgG, Fcγ fragment specific (1:5000 dilution ratio) was added to each well. After washing three times with PBST, 50 μL of TMB substrate solution was added. The mixture was incubated at room temperature in the dark for 10 min. 50 μL of 2 M H_2_SO_4_ was added to each well to stop the reaction. The absorbance of each well of the microplate was measured at 450 nm.

PD-1-hFc and VEGF-His were diluted to 0.5 μg/mL and 1 μg/mL in PBS, respectively. A total of 50 μL per well was added to the microplate. The 96-well high-binding plate was incubated at 5 ± 3 °C for at least 15 h. After coating, the plate was washed once with PBST, after which it was blocked with 1% BSA in PBS for 1 h, after washing three times with PBST. BsAbs (starting at a concentration of 18 μg/mL at a 1:3 dilution ratio) were serially diluted into 10 gradients and mixed with the recombinant human CD206 protein (His tag) at a concentration of 720 ng/mL in equal volumes. In parallel, it was mixed with the diluent in equal volumes as a control. The 96-well dilution plate was incubated at 37 °C for at least 1 h. The BsAb mixture and control (diluent) were added to each well of a 96-well high-binding plate at 50 μL per well. After 10 min, 50 μL of the prepared 0.6 μg/mL ligand protein PDL-1-mFc and 50 μL of the prepared 0.2 μg/mL ligand protein VEGFR^2^-ECD-mFc-bio were added to each well, respectively. After gentle mixing, the plate was incubated at 37 °C for 40 min. After washing three times with PBST, 50 μL of HRP-goat anti-human IgG and 50 μL of SA-HRP were added to each well at 37 °C for 40 min, respectively. After washing three times with PBST, 50 μL of TMB substrate solution was added. The mixture was incubated at room temperature in the dark for 10 min. 50 μL of 2 M H_2_SO_4_ was added to each well to stop the reaction. The absorbance of each well of the microplate was measured at 450 nm, respectively.

### 4.6. Data Analysis

The LC/MS data were acquired via Chromoleon 6.8 and analyzed with Biopharma Finder 5.2. The LC data were acquired by Empower. All the data were analyzed with GraphPad Prism 7.0 (GraphPad Prism, San Diego, CA, USA). The RLU value (y) and log-transformed concentrations of the sample or standard reference (x) were fitted via a four-parameter equation (y = (a − d)/[1 + (x/c)b] + d) to calculate the IC50 (half maximal inhibitory concentration) of the sample.

## 5. Conclusions

We successfully identified unexpected *O*-glycosylations of commercially available therapeutic BsAb products by combining HPLC-PAD and HCD-MS/MS database analysis. We identified *O*-xylosylation at Ser residues in the (G4S)4 linker of BsAbs. In addition, *O*-xylosylation was observed at Ser residues on the (G4S)4 linker peptide, as previously reported. G0F was the most abundant *N*-glycan (76%) by HILIC-HPLC analysis. In addition to the glycoforms G0-GlcNAc, G0F-GlcNAc, G0, G1F, and Man5 also presented a low abundance (<10%), exhibiting negligible effects on Fc region function. We utilized mannose receptor coincubation binding assays to evaluate the activity of BsAbs, and the results demonstrated that these *O*-xylosylation reactions do not impair the functional activity of BsAbs. However, BsAbs were firstly found to interact with mannose receptors in vitro. There are no means available to investigate the impact of *O*-xylosylations on its Fc region more deeply because it inherently lacks CDC and ADCC functions. However, if BsAbs possess Fc functions, *O*-xylosylations modifications and binding to the mannose receptor would inevitably affect CDC and ADCC activity. We want to emphasize the importance of performing detailed *O*-xylosylation analysis to increase our understanding of the effects of *O*-xylosylation in BsAbs. Under a risk-based regulatory approval framework for protein therapeutics, *O*-glycosylation modifications should be thoroughly characterized. This is particularly critical for ensuring the consistent quality, safety, and efficacy of the product.

## Figures and Tables

**Table 1:** LC conditions for intact protein, HC-LC chain and peptide mapping analysis.

	Intact Protein			HC-LC Analysis			Peptide Mapping		
Mobile phase			A: FA/water (1:1000, *v*/*v*); B: FA/acetonitrile (1:1000, *v*/*v*)						
Flow rate (mL/min)	0.3			0.3			0.2		
column temperature (°C)			60						
wavelength for UV	280 nm								
injection amount	2 µg			2 µg			25 µg		
gradient	Time (min)	A%	B%	Time (min)	A%	B%	Time (min)	A%	B%
	0	80	20	0	75	25	0	98	2
	15	68	32	15	60	40	5	98	2
	18	10	90	18	10	90	120	65	35
	20	10	90	20	10	90	128	0	100
	20.1	80	20	20.1	75	25	128.1	98	2
	25	80	20	25	75	25	135	98	2

**Table 2:** LC conditions for *N*-glycosylation.

	*N*-Glycosylation			
Mobile phase	A: 50 mmol/L ammonium formate solution (pH 4.5); B: 1.0 N Sodium Hydroxide and Acetonitrile			
column temperature (°C)	40			
Wavelength for Fluorescence	excitation at 330 nm and emission at 420 nm			
injection volume	1 µL			
gradient	Time (min)	Flow rate (mL/min)	A%	B%
	0	0.4	22	78
	10	0.4	22	78
	34.8	0.4	47	53
	36	0.25	80	20
	39	0.25	80	20
	40	0.25	22	78
	45	0.25	22	78
	45.1	0.4	22	78
	50	0.4	22	78

**Table 3:** LC conditions for *O*-glycosylation.

	*O*-Glycosylation			
Mobile phase	A: 50 mM Sodium Acetate; B: 1.0 N Sodium Hydroxide and 50 mM Sodium Acetate			
column temperature (°C)	40			
Detection	Pulsed Amperometric detection, gold working electrode, pH/Ag/AgCl reference			
injection volume	50 µL			
gradient	Time (min)	Flow rate (mL/min)	A%	B%
	0	0.4	80	20
	30	0.4	80	20
	30.1	0.4	35	65
	57	0.4	35	65
	57.1	0.4	80	20
	70	0.4	80	20

**Table 4:** MS parameters for intact protein, HC-LC chain and peptide mapping analysis.

	Intact Analysis	HC-LC Analysis	Peptide Mapping
Spray voltage (kV)	3.6	3.6	3.6
sheath gas (arb)	35	35	35
aux gas (arb)	10	10	10
gas heater temperature (°C)	350	350	350
capillary temperature (°C)	320	320	320
m/z for MS1	1000–4000	2000–8000	200–2000
isolation window (Da)	-	-	2
First mass for MS2	-	-	110
AGC (%) for MS1	500	300	300
Resolution for MS1	17,500	17,500	70,000
maximum injection time (ms) for MS1	200	100	100
AGC (%) for MS2	-	-	100
Resolution for MS2	-	-	17,500
maximum injection time (ms) for MS2	-	-	50
(N)CE (%)	-	-	27
top N	-	-	5
microscan	5	5	1

## Data Availability

Data is contained within the article.
